# P-1176. Contezolid in the Management of Mycobacterial Infections among Pediatric HSCT Recipients with Primary Immunodeficiency: A Retrospective Study

**DOI:** 10.1093/ofid/ofaf695.1369

**Published:** 2026-01-11

**Authors:** Ximu Sun, Wei Yang, Peng Guo, Maoquan Qin, Guanghua Zhu

**Affiliations:** Beijing Children's Hospital, Capital Medical University, National Center for Children’s Health, Beijing, Beijing, China (People's Republic); Beijing Children's Hospital, Capital Medical University, National Center for Children’s Health, Beijing, Beijing, China (People's Republic); Beijing Children's Hospital, Capital Medical University, National Center for Children's Health, Beijing, Beijing, China; Beijing Children's Hospital, Capital Medical University, National Center for Children’s Health, Beijing, Beijing, China (People's Republic); Beijing Children's Hospital, Capital Medical University, National Center for Children’s Health, Beijing, Beijing, China (People's Republic)

## Abstract

**Background:**

Children with primary immunodeficiency disease (PID) are at high risk of mycobacterial infections. Although linezolid (LZD) remains a mainstay of therapy, its utility is limited by hematologic toxicity, which is a critical concern in hematopoietic stem cell transplant (HSCT) recipients. Contezolid (CZD), a novel oxazolidinone antibiotic, has shown potent antibacterial-mycobacterial activity and an improved safety profile, offering a promising therapeutic option.

**Table 1:**
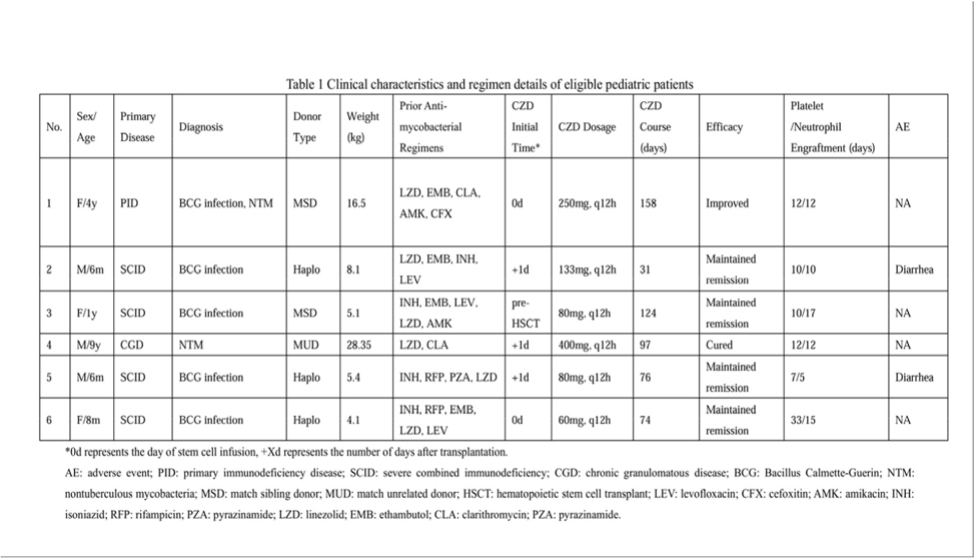
Clinical characteristics and regimen details of eligible pediatric patients

**Methods:**

We conducted a retrospective cohort study involving children with PID who underwent allo-HSCT at Beijing Children's Hospital from May 2023 to December 2024. Eligible patients were those with microbiologically confirmed tuberculosis (TB) and nontuberculous mycobacteria (NTM) infections who were treated with CZD following a weight-adjusted dosing protocol at approximately 16 mg/kg twice daily.

**Results:**

A total of 6 patients were included. The characteristics are shown in Table 1. These patients had negative NGS results prior to CZD initiation and maintained remission throughout the subsequent treatment course. The median treatment duration was 86.5 days (IQR: 74.5-117.25). Following myeloid reconstitution, clinicians transitioned most patients to regimens with more established pediatric evidence. However, Case 4, a 9-year-old boy with NTM, continued with the original treatment with CZD and demonstrated sustained clinical efficacy. The median follow-up period was 146 days (IQR: 144.25-330). One mortality occurred in Case 6 due to septic shock and pulmonary hemorrhage caused by mixed infection of multiple pathogens, accompanied by delayed platelet engraftment ( >1 month). Two cases of diarrhea were reported during CZD treatment, which were not considered to be related to CZD based on clinical judgment. No apparent liver or kidney disorder, neurotoxicity, or lactic acidosis was observed.

**Conclusion:**

This study suggests that CZD as a feasible bridging therapy for mycobacterial infection during the post-transplant aplasia period. It exhibited no significant myelosuppression or delayed platelet engraftment, effectively prevented infection progression, and demonstrated favorable tolerability.

**Disclosures:**

All Authors: No reported disclosures

